# Genome-Wide Analysis of the Malaria Parasite *Plasmodium falciparum* Isolates From Togo Reveals Selective Signals in Immune Selection-Related Antigen Genes

**DOI:** 10.3389/fimmu.2020.552698

**Published:** 2020-10-23

**Authors:** Kokouvi Kassegne, Komi Komi Koukoura, Hai-Mo Shen, Shen-Bo Chen, Hai-Tian Fu, Yong-Quan Chen, Xiao-Nong Zhou, Jun-Hu Chen, Yang Cheng

**Affiliations:** ^1^Laboratory of Pathogen Infection and Immunity, Department of Public Health and Preventive Medicine, Wuxi School of Medicine, Jiangnan University, Wuxi, China; ^2^Laboratoire des Sciences Biomédicales, Alimentaires et Santé Environnementale, Département des Analyses Biomédicales, Ecole Supérieure des Techniques Biologiques et Alimentaires, Université de Lomé, Lomé, Togo; ^3^National Institute of Parasitic Diseases, Chinese Centre for Disease Control and Prevention, Chinese Centre for Tropical Diseases Research, WHO Collaborating Centre for Tropical Diseases, National Centre for International Research on Tropical Diseases, Ministry of Science and Technology, Key Laboratory of Parasite and Vector Biology, Ministry of Health, Shanghai, China; ^4^National Institute of Parasitic Diseases, Chinese Centre for Disease Control and Prevention—Shenzhen Centre for Disease Control and Prevention Joint Laboratory for Imported Tropical Disease Control, Shanghai, China; ^5^The School of Global Health, Chinese Centre for Tropical Diseases Research, Shanghai JiaoTong University School of Medicine, Shanghai, China; ^6^School of Food Science and Technology, State Key Laboratory of Food Science and Technology, Jiangnan University, Wuxi, China

**Keywords:** *Plasmodium falciparum*, genomes, balancing selection, directional selection, immunity, Togo

## Abstract

Malaria is a public health concern worldwide, and Togo has proven to be no exception. Effective approaches to provide information on biological insights for disease elimination are therefore a research priority. Local selection on malaria pathogens is due to multiple factors including host immunity. We undertook genome-wide analysis of sequence variation on a sample of 10 *Plasmodium falciparum* (Pf) clinical isolates from Togo to identify local-specific signals of selection. Paired-end short-read sequences were mapped and aligned onto > 95% of the 3D7 Pf reference genome sequence in high fold coverage. Data on 266 963 single nucleotide polymorphisms were obtained, with average nucleotide diversity π = 1.79 × 10^−3^. Both principal component and neighbor-joining tree analyses showed that the Togo parasites clustered according to their geographic (Africa) origin. In addition, the average genome-wide diversity of Pf from Togo was much higher than that from other African samples. Tajima’s *D* value of the Togo isolates was −0.56, suggesting evidence of directional selection and/or recent population expansion. Against this background, within-population analyses identifying loci of balancing and recent positive selections evidenced that host immunity has been the major selective agent. Importantly, 87 and 296 parasite antigen genes with Tajima’s *D* values > 1 and in the top 1% haplotype scores, respectively, include a significant representation of membrane proteins at the merozoite stage that invaded red blood cells (RBCs) and parasitized RBCs surface proteins that play roles in immunoevasion, adhesion, or rosetting. This is consistent with expectations that elevated signals of selection due to allele-specific acquired immunity are likely to operate on antigenic targets. Collectively, our data suggest a recent expansion of Pf population in Togo and evidence strong host immune selection on membrane/surface antigens reflected in signals of balancing/positive selection of important gene loci. Findings from this study provide a fundamental basis to engage studies for effective malaria control in Togo.

## Introduction

Malaria clinical presentation ensues when *Plasmodium* parasites invade and destroy red blood cells (RBCs). Fever and chills occur at the time of rupture of infected RBCs (iRBCs) containing merozoites that are freed to invade uninfected RBCs (1, 2). Failure to receive prompt treatment may lead to dyserythropoietic anaemia or severe malaria. *P. falciparum* (Pf) is the most dangerous malaria parasite because of the high level of mortality with which it is associated, its widespread resistance to antimalarial medicines, and its dominance in the world’s most malarious continent, Africa (3–5).

In Togo, malaria transmission occurs most of each year. Although decades of control efforts have reduced the disease burden, the entire country’s population is still at risk of falciparum malaria infection (6). In addition, challenges in parasite control would have made the infection a public health concern and may aggravate the difficulty of treatment. Clinical spectrum of malaria in Togo usually ranges from asymptomatic carriage of malaria parasites to a febrile disease that may evolve into a severe, life-threatening illness, making the infection a major cause of morbidity and mortality, especially in children (7, 8). Antimalarial drug resistance (e.g., parasite resistance to chloroquine or pyrimethamine) has been experienced across Africa. In early investigations in Togo, clinical and parasitological therapeutic failure tests of artemether-lumefarine (AL) and artesunate-amodiaquine (ASAQ) for 3% and 3.8%, respectively, have been observed (6), and they drew the entire country’s attention to an eventual resistance to artemisinins. However, in a recent study, therapeutic efﬁcacy of **AL** and ASAQ was shown without delay in the clearance of mutant parasites (9). Pf surface-exposed proteins are targets of host immune responses, and repeated exposures to the parasite in endemic areas induce a slow and gradual development of acquired immunity to clinical malaria, which is usually evidenced as a decline in the prevalence of clinical episodes (10, 11). Hence, acquisition of information on both immunity-related antigens and drug resistance genes for effective interventions to sustain and drive forward the struggle against malaria parasite in Togo is therefore a research priority.

Complete sequencing of the Pf genome has boosted post-genomic studies of malaria (12). It provides fundamental knowledge for better understanding of the cellular and molecular mechanisms of infection and immunity to develop new control methods, including new drugs and vaccines, improved diagnostics, and effective vector control techniques. With rapid development of sequencing technologies (13), hundreds of falciparum isolate genomic data worldwide had been investigated and shared by large collaborative initiatives such as the MalariaGEN Pf Community Project and the Pf3k Consortium. Application of the genomic approaches in the analysis of whole genome variations–generated high-density single nucleotide polymorphisms (SNPs) of the parasite has mostly focused on vaccine antigen genes and drug-resistant genes. However, to date, nothing is known on genomes of malaria isolates in Togo, and this could limit the joint research with those in other endemic areas in the sub-Saharan Africa region.

In this study, we performed the first whole-genome sequencing (WGS) of Pf clinical isolates from Togo. With the aim to contribute to accelerating the pursuit of effective malaria control, we applied genomic approaches in the analysis of whole genome variations–generated high-density SNPs to provide biological insights on target genes, especially those under host immune selection.

## Materials and Methods

### Sampling Sites and Ethics Statement

**Figure 1:**
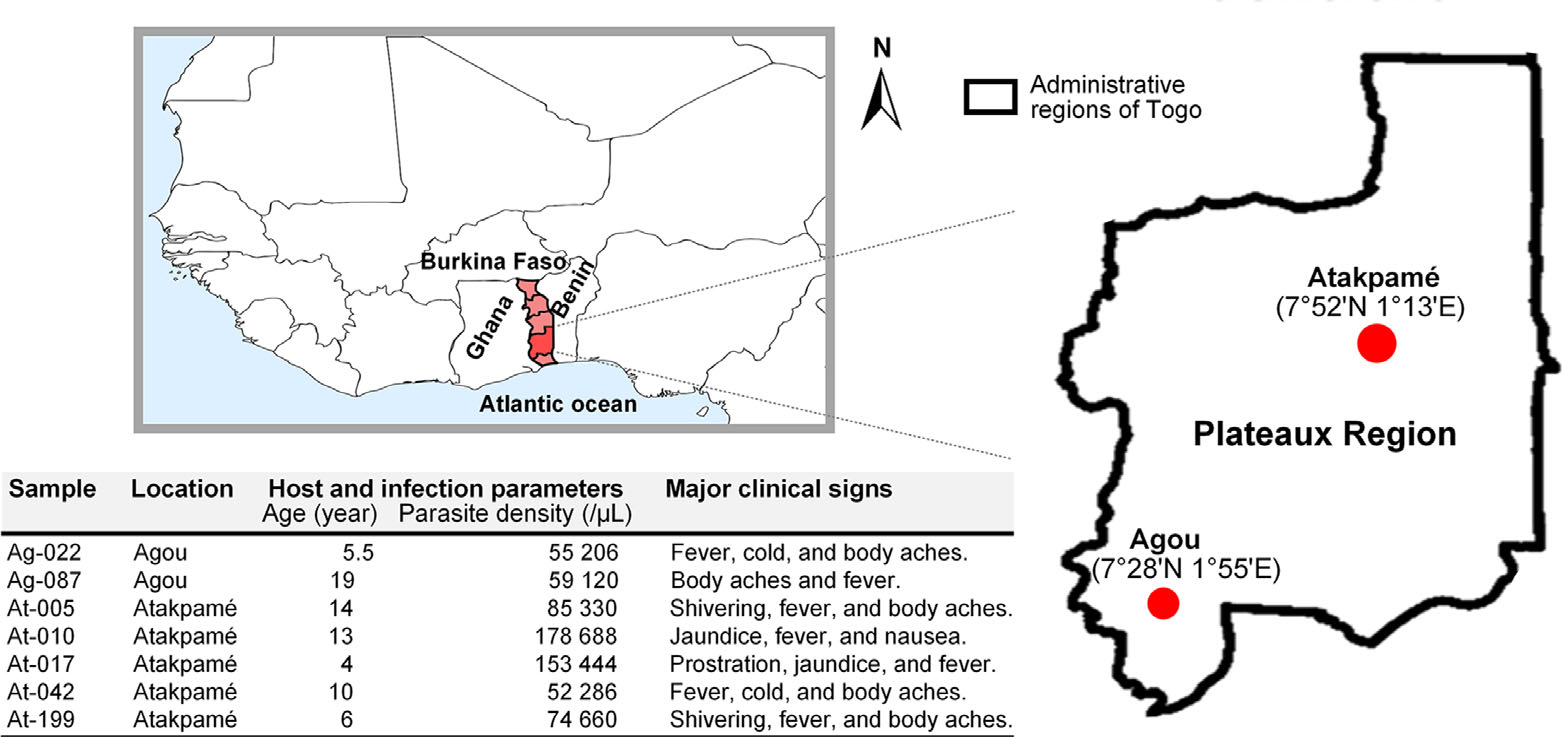
Sampling location in Togo and information about clinical samples used in this study.

Malaria transmission in Togo occurs for most of each year with seasonal outbreaks (9), and populations are served by health facilities experienced in the management of malaria cases. For this study, clinical samples were collected at health centres in urban areas of Agou-Gadzépé (7°28’01’’ N; 1°55’01’’ E) and Atakpamé (7°52’87’’ N; 1° 13’05’’ E) in Agou and Ogou prefectures, respectively, in the Plateaux Region (**Figure 1**) in 2017 and 2018. Samples collection was made under a study protocol approved by the Togolese Ministry of Health’s Bioethics Committee following institutional ethical guidelines by the ethics committee at National Institute of Parasitic Diseases, Chinese Centre for Disease Control and Prevention. Informed consent was obtained from all subjects prior to sample collection.

### Genomic Data

For our analyses, genome and annotation data of Pf 3D7 strain (the most complete whole genome standard reference) from PlasmoDB database (http://plasmodb.org/plasmo/) (14) were downloaded. In addition, raw sequences from 62 genome data of falciparum clinical isolates from Africa [n = 32 (Congo DR, Gambia, Ghana, Guinea, Malawi, Mali, Nigeria, and Senegal)] and Asia [n = 30 (Bangladesh, Cambodia, China-Myanmar Border (CMB), Laos, Myanmar, Thailand, and Viet Nam)] were also referenced (15–17).

### Sampling of Malaria Parasites and Extraction of Genomic DNA

Malaria-naturally exposed subjects who received parasitological diagnosis using Giemsa-stained thick blood smear microscopy under 1000x magnification were referred to our study. Whole blood specimens from subjects who were diagnosed with the presence of Pf asexual parasitaemia (parasites counted per 200 leukocytes and parasite density calculated as the number of parasites per microliter by assuming a fixed leukocyte count of 8000 cells/μL of blood) were sampled as dried blood spots (DBSs) on Whatman FTA cards (GE Healthcare) as recommended by the manufacturer. Genomic DNA was extracted [using the QIAGEN DNeasy Blood & Tissue Kit (Qiagen), according to the manufacturer’s instructions] from DBSs and monospecies infection was confirmed by polymerase chain reaction (PCR). Ten clinical samples with high parasitaemia (parasite density > 50000/μL), and qualitatively and quantitatively good enough, were selected to ensure the integrity of sequencing.

### Whole-Genome Sequencing

WGS of Pf clinical isolates from Togo was performed by OE Biotech (Shanghai). Extracted genomic DNA was sheared into 150 bp fragments using a Covaris instrument. The fragmented DNA molecules were used to construct Illumina-sequencing libraries with TruSeq DNA LT Sample Prep Kit (Illumina). All libraries were sequenced on the Illumina HiSeq X10 platform according to the manufacturer’s protocol (18), using the direct sequencing approach, as described previously (17). All reads were filtered by removing the adapter sequences and low quality sequences were removed with Trimmomatic-3.0. (19). The sequencing reads have been submitted to the Short Read Archive of the National Centre for Biotechnology Information.

### Identification of SNPs and Population Structure

All sequenced reads from the 10 samples were mapped to the Pf 3D7 genome using Burrows-Wheeler Aligner and Sequence Alignment/Map (SAMtools-1.3) (20). Samples with average coverage < 95% sequences mapping over 3D7 reference genome were removed. For high-quality SNP calling, sequencing reads were genotyped using an in-house pipeline based on GATK best practices and SnpEff workflows (21), with Pf3K known-sites (15).

Principal component analysis (PCA) and neighbor-joining were performed to investigate major geographical division of population structure. PCA and a neighbor-joining tree of all samples were undertaken via SPSS-Ver25 and Mega-Ver6.0 programs, respectively, to compare Pf SNPs from Togo isolates with those from the 62 isolates collected worldwide (15–17).

### Tests for Signatures of Selection

For SNPs in all populations, nucleotide diversity (π) was estimated for the whole genome mutation rate in 4 kb sliding window and 2 kb step across each chromosome in Arlequin-Ver3.5 (22). To distinguish between genes evolving neutrally and under selective pressures, or genetic hitchhiking, Tajima’s *D* value (TD) for each sliding window and the corresponding gene was also calculated.

In addition, long-range haplotype diversity approach integrated haplotype score (iHS) was employed to identify genes under recent positive selection. iHS compares integrated extended-haplotype homozygosity (EHH) values between alleles at a given SNP (23). iHS computation was based on the Togo clinical isolates by tracking the decay of haplotype homozygosity for both the ancestral and derived haplotypes extending from every SNP site (24). For this test, we restricted the analyses to SNPs with inferred ancestral states with minor allele frequencies equal to or higher than 5% (25). iHS scores were estimated using Selscan-Ver1.10a (26).

To assess whether genes associated with putative functions were enriched among the group of genes with high Tajima’s *D* values (> 1.0) or high |iHS| (top 1% score), gene ontology (GO) term analysis was conducted. Genes with a TD > 1.0 were classed as genes of potential interest for GO analysis. Analysis was performed using GO Enrichment tool of PlasmoDB (http://plasmodb.org/plasmo/, PlasmoDB Ver-46). The adjusted *P* values were also generated from Fisher’s exact test, and the statistical significance was set for *P* < 0.05.

## Results

### Genetic Diversity of *Falciparum* Isolates From Togo

We used a direct sequencing approach that requires only high parasitaemia for malaria parasites without leukocytes filtration (17) to sequence clinical isolates of Pf genomes from Togo. Among the 10 clinical samples that were sequenced, results of seven were good enough and provided enough coverage (> 95% sequences mapping over the 3D7 reference genome) (**Table 1**). The remaining three samples mapped onto only 58.89%, 56.43%, and 46.83% (unshown data) and failed for further analysis. In this study, the Togo isolates generated between 55 and 176 M paired-end reads of 150 bp from each of the samples, globally. All sequencing reads have been deposited to the National Centre for Biotechnology Information (NCBI) Short Read Archive (Bio-Project Accession Number: PRJNA616298). A variable proportion of reads (3.8–14.2%) from all the isolates were mapped to the reference and aligned onto at least 95% of the reference 3D7 strain genome in high fold coverage (7.2–33.9x).

**Table 1:** Sequencing and mapping summary of Pf genome of seven clinical isolates from Togo.

Samples	Ag-022	Ag-087	At-005	At-010	At-017	At-042	At-199
**Sequencing and mapping**							
Number of clean reads	95 290 916	56 567 466	55 109 050	135 016 594	132 741 868	141 629 208	176 447 420
Mapped on Pf	3 796 286	3 414 970	7 700 867	6 078 064	5 761 033	6 713 431	6 615 316
Mapped (%)	4.0	6.1	14.2	4.5	4.4	4.8	3.8
**Coverage**							
Coverage fold	7.2	10.7	33.9	13.1	13.4	13.5	11.8
Genome covered >1 (%)	95.3	97.7	98.7	97.6	98.3	97.8	98.1
**Variation**							
Filtered SNP	26 091	35 139	57 129	37 065	37 828	35 653	38 058

**Figure 2:**
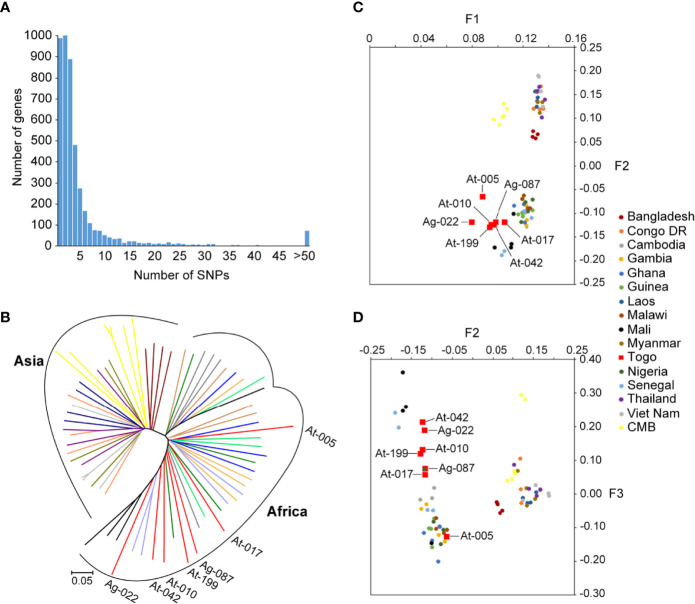
SNPs frequency distribution in samples and genomic relationships among Pf reference strains and Togo isolates. **(A)** Distribution numbers of genes with each given number of SNPs in the population sample of seven Pf clinical isolates from Togo. From the 4614 genes analyzed in total from 14 chromosomes, 20.2% (931/4614) had more than five SNPs. **(B)** Neighbor-joining tree of Pf constructed from the SNPs occurring in at least half of the samples. Lineages are colored according to geographic origin. Branch lengths indicate considerable diversity in Pf strains. Annotated branches represent the Togo isolates. **(C, D)** Principal component analysis based on common SNP loci in Togo clinical isolates and reference strains. Colors correspond to the geographic origin of the samples, of which the Togo isolates are highlighted in red. **(C)** The major fact (F1) of differentiation of the PCA identified clearly the two groups of isolates that clustered according to their geographic origin. **(D)** The second and third facts (F2 and F3) defined a distinct South-Asia cluster and distinguished the African samples better according to their locations.

For analysis of polymorphism, a total of 266963 SNPs common loci were available for analysis after quality filtering (**Table 1**). The list of the SNPs for all the isolates is provided in **Supplementary Table 1**. Of the 266963 SNPs, excluding the low-frequency SNPs (103497 SNPs with minor allele frequency < 5%), a total of 163466 SNPs across the seven isolates were identified and could be mapped to coding sequences. In addition, SNPs were identified across 4614 genes on 14 chromosomes in the samples and 931 genes had more than five SNPs (**Figure 2A**). These genes were considered informative for comparisons of polymorphic nucleotide sites.

### Comparison of Genetic Diversity of the Isolates Among Different Endemic Regions

Overall genome-wide π of Pf clinical isolates from Togo were estimated at 1.79 × 10^−3^. However, genetic diversity was lower in intronic regions but higher in exonic and intergenic regions (**Supplementary Table 1**). **Supplementary Figure 1** shows the π map of the isolates across 14 chromosomes. Interestingly, we observed that Togo samples have genes with higher SNPs, suggesting a greater genetic diversity than that reported from other African samples (π = 1.03 × 10^−3^) (27), but lower than that of isolates from CMB (π = 2.87 × 10^−2^) (17).

We then performed PCA and neighbor-joining analyses of all strains to assess major geographical difference. As part of Africa isolates, the Togo isolates illustrated a higher discrepancy than the 3D7 strain genome. Neighbor-joining displayed a tree with two distinct branches separating two major clades that correspond to the Asia and Africa geographical groups of samples (**Figure 2B**). There was evidence of clear distinction of the isolates from the two regions, and African isolates displayed sub-clusters to form two (or three) monophyletic clades. Furthermore, we found that the outcome from PCA was similar to that of the neighbor-joining analysis. The major axis of differentiation (F1) of the PCA distinguished clearly two major Asia and Africa groups of isolates, which is in accordance with their geographical origins (**Figure 2C**). Similar observation was noted in recent studies on Pf isolates from CMB (17, 28). In addition, among the Africa samples, Togo samples exhibited greater genetic diversity than has been reported from other African regions. The second and third principal components (F2 and F3) defined a distinct South-Asian cluster and distinguished the African samples better according to their locations, where Togo samples were well differentiated from other African samples (**Figure 2D**). Furthermore, Togo isolates were widely separated in our PCA result, suggesting high diversity of Pf from Togo.

### Signatures of Selection in the Isolates From Togo

**Figure 3:**
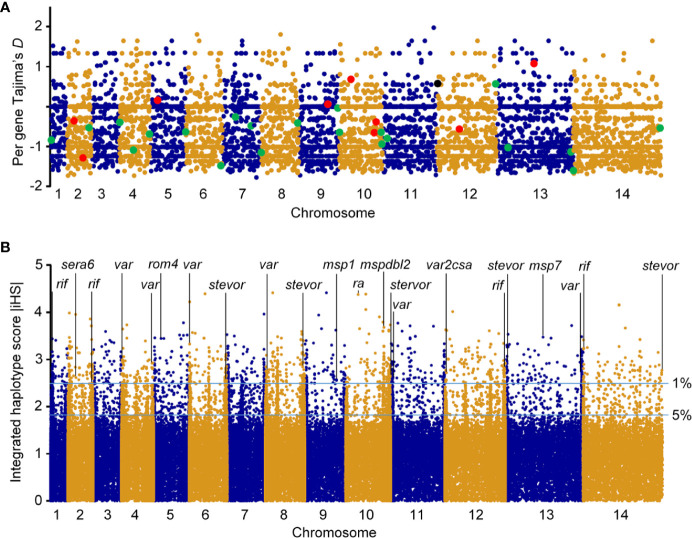
Genomic map of Pf isolates from Togo. Paired-end short-read sequencing produced high-quality data for a population sample of seven falciparum clinical isolates from Togo, with genome-wide average mapping depth to the 3D7 reference strain genome. **(A)** Tajima’s *D* values map of falciparum clinical isolates from Togo. Values for all gene SNPs were plotted and arranged according to their chromosomal positions (blue and gold colors indicate consecutive chromosomes numbered from the smallest upwards). TD for RBC invasion-related antigen genes are shown in enlarged red dyes and those for genes implicated in parasite-mediate immunoevasion, cytoadhesion, resetting/sequestration, or deformability of RBC/rigidity, are indicated in enlarged green dyes. Pregnancy malaria-related *var2csa* is also highlighted (black dye). **(B)** Top |iHS| hits in Pf isolates from Togo with SNPs minor allele frequency ⩾ 5%. *x* axis indicates individual chromosomes in alternating colors of their SNPs; *y* axis is the value of |iHS|. Plot of genome-wide |iHS| scores shows regions of the genome that have windows of elevated values with high scoring (top 1% of |iHS| values) for important gene loci highlighted, consistent with the operation of recent positive directional selection. The horizontal lines represent values of 2.49223 and 1.8157 used to define windows containing SNPs with overlapping regions of EHH.

We investigated signatures of selection of the parasite in this sub-Saharan Africa region. TD of the Togo isolates was −0.56 across the entire genome (**Figure 3A**), indicating a population history of purifying selection and/or recent population expansion. To study allele frequency distributions for individual genes, with the gene transfer format file containing information about gene structure, we annotated genes and then calculated TD for the individual genes. Of the 5601 genes analyzed, the TD obtained were mostly negative (3759 genes, average TD = -0.95). Such predominantly negative values are consistent with previous analyses indicating a historical population expansion of Pf in Africa (29), and it suggests that these genes were under selective sweep (directional selection) (17, 28, 30). We found 931 genes that each had at least five SNPs. A list of the top 250 lowest values for genes with at least one SNP (n = 4614) is provided in **Supplementary Table 2**. Against this background, 746/4614 genes (16.2%) had positive TD values (**Supplementary Table 3**), of which 140 genes had values > 1 (87 genes coding proteins with known functions), suggesting signals of balancing selection for these genes (28, 31).

Mean pairwise divergence was higher in a significant representation of genes that encode membrane proteins expressed at the merozoite stage that invades RBCs (for example, merozoite surface proteins, MSPs; serine repeat antigens, SERAs; rhomboid proteases, ROMs; duffy binding-like merozoite surface proteins, MSPDBLs; rhoptry associated adhesins, RA) (32, 33), and parasitized RBC surface proteins that play roles in disease severity—immune evasion, rosetting, or cytoadherence to microvasculature (repetitive interspersed family of polypeptides, RIFINs; erythrocyte membrane protein 1, PfEMP1; and subtelomeric variant open reading frames, STEVORs) (34–37). Importantly, there was evidence of balancing selection on particular genes including antigen genes related to RBC invasion, including those with solid balancing selection (reflected in high TD) [*sera5* (TD = 1.42); apical asparagine-rich protein, *aarp* (TD = 1.34); ferlin-like protein, *flp* (TD=1.29); *msp3* (TD = 1.28); and *msp7* (TD = 1.08)] (**Table 2**) and those with TD < 1 [phospholipase, *pl*; erythrocyte binding antigen-175, *eba175*; reticulocyte binding protein 2 homologue a, *rh2a*; *ra*; 6-cysteine protein, *pf41*; apical membrane antigen 1, *ama1*; *sera4*; glutamate-rich protein, *glurp*; merozoite TRAP-like protein, *mtrap*; *rom4*; membrane associated erythrocyte binding-like protein, *maebel*; rhoptry neck protein 2, *ron2*; subtilisin-like protease 1, *sub1*; and *msp1*] (**Supplementary Table 3**). Most of these antigen genes were reported previously for the balancing selection (28, 31, 38, 39) and were significantly enriched by GO analysis (*P* < 0.0001). Although antigen genes associated with parasite-mediated immune evasion, adhesion, or rosetting were found in the positive TD list [13 *rifs* (PF3D7_1000600, PF3D7_1254800, PF3D7_0713000, PF3D7_0632100, PF3D7_1040900, PF3D7_1300400, PF3D7_1254700, PF3D7_1150300, PF3D7_0808800, PF3D7_0114700, PF3D7_1101300, PF3D7_1100300, and PF3D7_0401300), three *vars* (PF3D7_0302300, PF3D7_1200600, and PF3D7_0601400) and one *stevor* (PF3D7_1479900)] (**Supplementary Table 3**) and were all highly significantly enriched by GO analysis (*P* < 0.0001), only the *var* PF3D7_0302300 is likely under strong balancing selection (TD = 1.33) (**Table 2**). Interestingly, PF3D7_1200600 (TD = 0.57) is the Pf *var* gene (*var2csa*) implicated in pregnancy malaria (34). GO analysis showed significant drug resistance enrichment for amino acid transporter *aat1* and bifunctional farnesyl/geranylgeranyl diphosphate synthase *fpps*/*ggpps*, which got TD > 1.

**Table 2:** Ten Pf genes with Tajima’s *D* scores > 1 enriched by GO analysis in Togo isolates.

PlasmoDB accession number	Product description	Genomic location	Tajima’s *D*
PF3D7_1128400	bifunctional farnesyl/geranylgeranyl diphosphate synthase, FPPS/GGPPS	Chr11: 1104216 - 1106505 (-)	1.64955
PF3D7_0207600	serine repeat antigen 5, SERA5	Chr02: 303593 - 307027 (-)	1.42303
PF3D7_0423400	apical asparagine-rich protein, AARP	Chr04: 1055665 - 1056318 (+)	1.34164
PF3D7_0302300	erythrocyte membrane protein 1 (PfEMP1), pseudogene	Chr03: 125992 - 130,235(-)	1.32775
PF3D7_0806300	ferlin-like protein, putative, FL	Chr08: 337902 - 343,254 (-)	1.28799
PF3D7_1035400	merozoite surface protein 3, MSP3	Chr10: 1404195 - 1405259 (+)	1.27765
PF3D7_0201600	PHISTb* domain-containing RESA-like protein 1, PHISTb RLP1	Chr02: 77251 - 78808 (-)	1.25357
PF3D7_0629500	amino acid transporter, AAT1	Chr06: 1213948 - 1216005 (-)	1.16843
PF3D7_1335100	merozoite surface protein 7, MSP7	Chr13: 1419086 - 1420141 (-)	1.07565
PF3D7_0629300	phospholipase, putative, PL	Chr13: 1205190 - 1207781 (+)	1.00902

*Plasmodium helical interspersed subtelomeric b.

Antigenic variation within Pf surface-exposed putative proteins is a target of host immune selection. Therefore, we applied iHS for all SNPs from Pf isolate genomes to investigate genome-wide evidence for positive selection (**Figure 3B**). We identified all 14 chromosomal regions with loci above the top 5% value (|iHS| > 1.8157) of the randomly expected distribution including 646 genes (**Supplementary Table 4**). Using |iHS| = 2.49223 (top 1% expected distribution) as a strong hits threshold, we identified 306 genes under significant positive selection (38, 40), of which 296 had at least five SNPs (**Supplementary Table 5**).

This analysis identified the selection signals for important genes with loci above the top 1% iHS score (|iHS| > 2.49223), including 10 RBC invasion-related antigen genes [*msp1*, *msp4*, *msp7*, *msp9*, *ron2*, *mspdbl1*, *mspdbl2*, *ra*, *sera6*, and *rom4*] (**Supplementary Table 6**). From these, *msp1*, *msp7*, *mspdbl1*, *mspdbl2*, *ra*, and *sera6* were highly significantly enriched by GO analysis (*P* < 0.0001) (**Table 3**) and have been reported previously as promising subunit candidates for a malaria multicomponent vaccine (32, 39, 41). Similarly, 134 genes implicated in roles for immune evasion, RBCs aggregation, or cytoadherence to microvasculature (73 *rifs*, 50 *vars*, and 12 *stevors*) were identified (**Supplementary Table 7**), of which six *rifs*, seven *vars*, and eight *stevors* (**Table 3**) were reported previously as targets of acquired immunity and may serve to prevent severe malaria (42–46). Furthermore, the *var2csa* that is implicated in pregnancy placental malaria (34, 47) was also observed in the top highest haplotype scores. Overall, iHS values aligned onto those obtained from Tajima’s *D* analysis. We also found signals of positive selection in genes that may be related to drug resistance (n = 8) within the top 1% iHS (**Supplementary Table 8**). From these, two genes (abc transporter I family member 1, *abcI3* and AP2 domain transcription factor, *apiap2*) (**Table 3**) were significantly enriched by GO analysis (*P* < 0.001) and were previously reported (48, 49). But, no selection signals were observed around the five known Pf drug resistance genes that include the chloroquine resistance transporter (*crt*), multidrug resistance-1 (*mdr1*), dihydrofolate reductase (*dhfr*), dihydropteroate synthase (*dhps*), and kelch 13 (*k13*).

**Table 3:** List of important Pf top 1% |iHS|-related genes enriched by GO analysis in Togo isolates.

PlasmoDB accession number	Product description	Genomic location	Core SNP position	|iHS|	TD
**RBC invasion**					
PF3D7_0207500	SERA6	Chr02: 298897 - 302564 (-)	302491	2.61685	-1.26953
PF3D7_0930300	MSP1	Chr09: 1201812 - 1206974 (+)	1202025	2.56848	0.01
PF3D7_1012200	RA	Chr10: 470979 - 471933 (+)	471579	4.37817	0.64916
PF3D7_1035700	MSPDBL1	Chr10: 1413200 - 1415293 (+)	1414316	2.90723	-0.38036
PF3D7_1036300	MSPDBL2	Chr10: 1432498 - 1434786 (+)	1434091	3.68371	-0.67735
PF3D7_1335100	MSP7	Chr13: 1419086 - 1420141 (-)	1419448	3.47128	1.07565
Disease severity*					
PF3D7_0100200	RIFIN	Chr01: 38982 - 40207 (-)	39702	3.16652	-0.84159
PF3D7_0223100	RIFIN	Chr02: 904551 - 905775 (+)	905045	2.4995	-0.58835
PF3D7_1040300	RIFIN	Chr10: 1609063 - 1610422 (+)	1610067	2.57465	-0.64439
PF3D7_1041100	RIFIN	Chr10: 1635596 - 1636779 (+)	1636505	2.76899	-0.93846
PF3D7_1254800	RIFIN	Chr12: 2228632 - 2229740 (-)	2229043	2.89538	0.56703
PF3D7_1400600	RIFIN	Chr14: 20897 - 22232 (-)	21300	3.23468	-1.60369
PF3D7_0400400	PfEMP1	Chr04: 45555 - 56860 (-)	45589	2.98694	-0.39466
PF3D7_0412700	PfEMP1	Chr04: 561667 - 569342 (-)	567264	2.67708	-1.12228
PF3D7_0425800	PfEMP1	Chr04: 1156423 - 1167821 (+)	1167689	2.92778	-0.69117
PF3D7_0600200	PfEMP1	Chr06: 3503 - 12835 (+)	4675	3.13913	-0.63996
PF3D7_0800300	PfEMP1	Chr08: 40948 - 50939 (+)	47670	2.76534	-1.15258
PF3D7_1100200	PfEMP1	Chr11: 32666 - 42386 (-)	38595	2.62163	-0.78851
PF3D7_1300300	PfEMP1	Chr13: 33959 - 44742 (-)	38488	2.71192	-1.03834
PF3D7_0631900	STEVOR	Chr06: 1333013 - 1334035 (+)	1333642	2.61356	-1.48024
PF3D7_0700400	STEVOR	Chr07: 36922 - 37927 (-)	37825	2.52706	-0.47579
PF3D7_0732000	STEVOR	Chr07: 1385635 - 1386626 (+)	1386482	2.77924	-0.27519
PF3D7_0832600	STEVOR	Chr08: 1405835 - 1406999 (-)	1406589	2.84218	-0.41204
PF3D7_0900900	STEVOR	Chr09: 55074 - 56081 (-)	55173	2.63821	-0.01639
PF3D7_1040200	STEVOR	Chr10: 1605930 - 1606953 (+)	1606790	2.70068	-0.65842
PF3D7_1300900	STEVOR	Chr13: 62515 - 63547 (-)	62808	2.96459	-1.13878
PF3D7_1479500	STEVOR	Chr14: 3269494 - 3270496 (+)	3270203	2.77831	-0.53876
**Pregnancy malaria**					
PF3D7_0201600	PHISTb RLP1	Chr02: 77251 - 78808 (-)	78077	3.37438	1.25357
PF3D7_1200600*	VAR2CSA	Chr12: 46788 - 56805 (-)	53438	2.71766	0.57512
**Drug resistance**					
PF3D7_0319700	ABCI3	Chr03: 820708 - 830802 (+)	821301	2.76481	-1.199
PF3D7_0613800	ApiAP2	Chr06: 566139 - 578993 (+)	571916	2.742	-0.280

*Antigen genes implicated in parasite-mediate immune evasion, deformability of RBC/rigidity of iRBC membrane, rosetting/sequestration, and/or cytoadhesion.

## Discussion

*P. falciparum* originated in Africa and spread to other continents as human migration gradually formed new populations (29). In this study, both the PCA and neighbor-joining tree analyses showed that the parasites derived from Togo clustered according to their geographic origin and distinguished two major clades that correspond to the Asia and Africa geographical groups of samples (17, 38). In addition, our data revealed the average nucleotide diversity of Pf from Togo is much higher than that from other African samples, but it is lower than the parasite from the CMB, probably due to the historically different antimalarial drugs used in that area (17). However, locally varying selection on pathogens due to differences in host immunity may be the major factor for the high nucleotide diversity observed in Togo isolates in comparison to other Africa isolates.

The purpose of the Tajima test is to detect deviation from neutrality, in other words, to indicate processes such as balancing selection, selective sweeps, and population expansion. This study revealed that some particular antigen genes that are related to RBC invasion and disease severity, and known to be polymorphic and under balancing selection by host immune system (31, 39), got TD < 0; suggesting selective sweep (directional selection) and/or recent population expansion. Interestingly, previous scans for evidence of positive selection on Pf have clearly identified loci that have undergone selective sweeps (38, 49, 50) as well as loci that are apparently under balancing selection, including those encoding targets of acquired immunity (31). In addition, some other investigations have observed multiple genes under recent positive selection by computation of iHS in other parasite populations (39, 40, 51, 52). Therefore, here, we applied iHS as a complementary analysis to assess signals of host immune selection.

In Pf isolates from Togo, within genes that are likely under signals of recent positive selection, host immunity-related antigen genes have been the major selective agents. In terms of the top outlier genes (top 1% |iHS| as a strong hits threshold and GO enrichment analysis), 31 of the 306 genes with known functions included six RBC invasion-linked antigen genes (*msp1*, *msp7*, *mspdbl1*, *mspdbl2*, *ra*, and *sera6*) (32, 41) and 22 antigen genes (six *rifs*, seven *vars*, and eight *stevors*) that are associated with roles in evasion to host immunity, rosetting or cytoadhesion (35–37), among which is *var2csa*, a pregnancy placental malaria-related gene (34, 47) (**Table 3**). Potential interest for GO analysis for genes under balancing selection by host immune system revealed six genes related to RBC invasion (*aarp*, *flp*, *msp3*, *msp7*, *pl*, and *sera5*) (32), one *var* (PF3D7_0302300) associated with pathogenesis (GO: 0009405), and *phistb rpl1* that is implicated in placental cytoadherence to microvasculature (47).

Interestingly, we found that most of the gene family members with elevated |iHS| are located close to each other on the chromosome. For example, from three *sera* genes that are contiguously arranged on chromosome two, *sera6* was involved in the top 1% SNP locus (|iHS| = 2.61685) and the remaining other two were also included in the 5% iHS list. This was also observed on chromosome two between *mps4* involved in the top 1% |iHS| (|iHS| = 2.83572) and *msp2* included in the 5% |iHS| (|iHS| = 2.08998). Following similar observation with eight serine-repeat antigen genes in *P. vivax* isolates (25), this could be explained by the process of positive natural selection increasing the prevalence of both selected variant as well as of nearby variants, generating local regions of extended haplotypes.

We identified genes that are likely to have been under exceptionally strong recent positive selection. Given these genes encode membrane/surface proteins, they would have been under high selection from the host immune system as potential selective targets of host immunity, and this may explain the high iHS scores that we observed (39, 41, 42). For example, highly elevated |iHS| associated with the gene encoding the MSP1 antigen was consistent with that from a previous report on Pf isolates from Gambia and Guinea, as this gene has a complex pattern of polymorphism that is likely to result from different selective processes (38). The MSP1, a core member of band 3 co-ligand complex during RBC invasion (32), has been validated as one of the leading blood-stage malaria vaccine antigens with sequences incorporated in experimental vaccine trials (41). In addition, highly supported windows of elevated iHS scores were also observed on chromosomes two and 10, incorporating the *sera6* and a cluster of different antigen genes (including *ra*, *mspdbl1*, and *mspdbl2*), respectively. Similarly, genes under high operation of positive selection in the Togo isolates include those encoding known surface antigens such as *vars* (PF3D7_1100200, PF3D7_0425800, PF3D7_1300300, and PF3D7_0400400) and promising targets of immunity that require further studies [members of *rif* (PF3D7_0223100, PF3D7_1400600, and PF3D7_0100200) and *stevor* (PF3D7_1040200, PF3D7_0631900, PF3D7_1300900, and PF3D7_0832600) families]. They are known to bind to cerebral endothelial/RBC surface receptors and have been identified or reported previously as immune targets that may serve to prevent severe malaria (43–45, 53, 54).

This analysis failed to detect selection signals for some important antigen genes such as *lsa3*, *ama1*, *msp2*, *msp3*, *eba175*, or circumsporozoite protein, *csp* [which have been entered vaccine-stage development (39, 41)], and *rif* (PF3D7_0100400, PF3D7_0401600, or PF3D7_1254800), *vars* (PF3D7_1150400, PF3D7_0533100, or PF3D7_0412700), or *stevors* (PF3D7_1254100 or PF3D7_0300400), to mention a few (43, 44, 53, 55), which have been identified or validated as targets of acquired immunity for vaccine development (39, 42). The reason could be that iHS may not be suitable for detecting positive selection for those SNPs that have reached fixation in a local population (28). Another possible explanation could be that they may be less targeted by host immunity in Togo subjects, given malaria transmission intensity and parasite genetic diversity are known to vary greatly among different parts of Africa due to variation in rainfall abundance and seasonality (39). However, immunological investigations using higher numbers of samples are needed in the future.

Positively skewed allele frequency distributions indicating the operation of balancing selection of Pf genes in other parasite populations have been reported (31, 38, 39, 56). In this study, the *phistb rlp1* encoding PHISTb domain-containing RESA-like protein 1 at the surface of iRBCs, which was reported previously as most likely under balancing selection (31, 38), was also identified. It interacts with VAR2CSA and modulates knob-associated heat-shock protein 40 expression on the iRBC surface, and thus may regulate VAR2CSA expression to confer stable chondroitin sulfate A binding capacity and the parasite’s cytoadherence (47). The *var2csa* was also detected among genes under strong positive selection in the Togo isolates. It encodes a particular parasite adhesion molecule (PfEMP1) expressed on the surface of iRBCs for roles in sequestration of Pf-iRBCs in the placenta, which occurs as a result of its binding to host receptors such as chondroitin sulphate A. Signals of strong balancing selection were evident in a similar subset of genes in Togo and other West Africa isolates. This is consistent with expectations that balancing selection due to allele frequency-dependent acquired immune responses is likely to operate on antigenic targets in Togo subjects (38). Such evidence could lead to studies for a vaccine to induce antibodies to prevent placental adhesion/sequestration by reducing the maternal anaemia and infant deaths that are associated with malaria in pregnancy (34, 39).

Furthermore, we found high |iHS| for two particularly important antigen genes (*msp7* and *phistb rlp1*), although they appear to being under balancing selection. The *msp7* in association with *msp1*, is important in invasion of mature RBCs and has been reported as a potential target of acquired immunity (32). Following similar observation with *csp* gene in *P. knowlesi* isolates (30), these genes could be targets of both balancing and directional selection due to their location within an elevated window of haplotype homozygosity on chromosomes, or might have hitchhiked to intermediate allele frequencies by a linked locus under selection within population-specific isolates.

Of the eight Pf drug-resistant genes identified within elevated iHS regions in Togo samples, none of the five known drug resistance genes (*crt*, *mdr1*, *dhfr*, *dhps*, and *k13*) were included, suggesting that Togo population is not under important antimalarial drug selection. This is consistent with a recent study in Togo that has shown therapeutic efﬁcacy of AL and ASAQ without delay in the clearance of mutant parasites (9). However, GO analysis for the drug-resistant genes that we identified by iHS computation within the top 1% |iHS| (*abcI3* and *apiap2*) or with TD > 1 (*aat1* and *fpps*/*ggpps*) were highly significantly (*P* < 0.001) enriched. In addition, our study suggested additional drug resistance genes under strong positive selection (**Supplementary Table 8**), which have been reported previously (48, 49).

## Conclusion

This study assessed the first whole-genome sequences of Pf isolates from Togo. Our results showed that the parasites derived from Togo clustered according to their geographic origin and suggest greater genetic diversity of Pf isolates in Togo than seen in other African countries. In addition, Tajima’s *D* values were predominantly negative, consistent with directional selection and/or a history of recent expansion of Pf population in Togo. Against this background, there was evidence of balancing and positive selections on particular genes. Loci showing evidence of recent positive selection and balancing selection attest that host immunity has been the major selective agent. This is reflected in a significant representation of genes that encode membrane proteins expressed at the merozoite stage that invades RBCs and parasitized RBC surface proteins implicated in roles for immunoevasion, rosetting, or cytoadhesion. Our study would contribute with insightful information on the current epidemiological scenario of malaria in Togo and provides a fundamental basis to engage studies for effective malaria control in Togo.

## Data Availability Statement

The datasets presented in this study can be found in online repositories. The names of the repository/repositories and accession number(s) can be found below: https://www.ncbi.nlm.nih.gov/, PRJNA616298.

## Ethics Statement

Permission was obtained from all malaria subjects before collecting specimens. Blood collection was made with informed consent from all individuals or their parents, under a study protocol reviewed and approved by the Togo Ministry of Health’s Bioethics Committee (Authorisation N°019/2019/MSHP/CBRS), following institutional ethical guidelines by the ethics committee at National Institute of Parasitic Diseases, Chinese Centre for Disease Control and Prevention.

## Author Contributions

KK, J-HC, and YC conceptualized the study. KK and KKK collected and analyzed the specimens. H-MC performed bioinformatics analysis. S-BC and H-TF participated in the experiments. KK interpreted the data and wrote the manuscript. KK, Y-QC, and Z-NZ revised the manuscript critically for intellectual content. All authors contributed to the article and approved the submitted version.

## Funding

This work was financially supported in part by the National Research and Development Plan of China (Grant No. 2018YFE0121600 and 2016YFC1202000), the National Natural Science Foundation of China (Grant No. 81601787, 81871681), the Natural Science Foundation of Jiangsu Province (Grant No. BK20160192), the Fundamental Research Funds for the Central Universities funded by the Ministry of Education of China (Grant No. JUSRP51710A), the Bill & Melinda Gates Foundation (Grant No. OPP1161962), the National First-Class Discipline Program of Food Science and Technology (Grant No. JUFSTR20180101), the Scientific Research Project of Public Health Research Centre of Jiangnan University (Grant No. 1285210162190530), and the Project of Shanghai Science and Technology Commission (Grant No. 18490741100). The sponsor played no roles in the study design or in the collection, analysis, or interpretation of the data, in writing the report, or in the decision to submit the article for publication.

## Conflict of Interest

The authors declare that the research was conducted in the absence of any commercial or financial relationships that could be construed as a potential conflict of interest.
