# Supplementary figures and images for: Genome-Wide Analysis of the Malaria Parasite Plasmodium falciparum Isolates From Togo Reveals Selective Signals in Immune Selection-Related Antigen Genes
Source: Front Immunol. 2020 Oct 23;11:552698. doi: 10.3389/fimmu.2020.552698 (PMC7645038; doi:10.3389/fimmu.2020.552698)

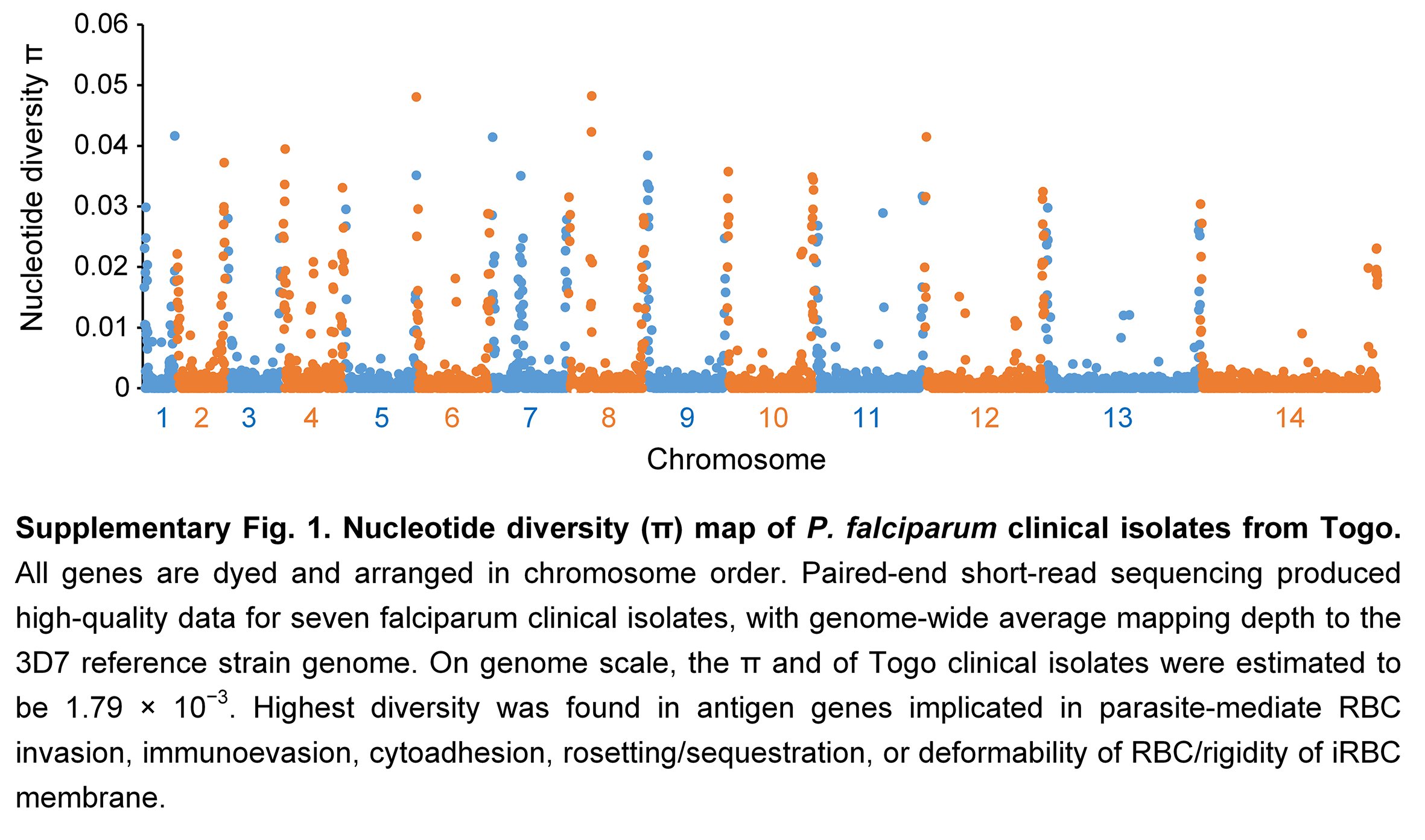

Supplement: Supplementary file 1 [file Image_1.tif]
